# Risk Factor of Posthemorrhagic Hydrocephalus: Cerebrospinal Fluid Total Protein

**DOI:** 10.3389/fsurg.2022.692383

**Published:** 2022-02-17

**Authors:** Zhiwen Wang, Yuxin Chen, Xinhui Zhou, Changfeng Wang, Xianjun Chen, Feixiang Min, Ruen Liu, Hui Xiang

**Affiliations:** ^1^Department of Neurosurgery, Jiangxi Provincial People's Hospital Affiliated to Nanchang University, Nanchang, China; ^2^Department of Geriatric Medicine, The Second Affiliated Hospital of Nanchang University, Nanchang, China; ^3^Department of Neurosurgery, The First Affiliated Hospital of Nanchang University, Nanchang, China; ^4^Department of Neurosurgery, Peking University People's Hospital, Beijing, China

**Keywords:** cerebrospinal fluid total protein, GCS, intraventricular hemorrhage, intracerebral hemorrhage, subarachnoid space hemorrhage, traumatic brain injuries, posthemorrhagic hydrocephalus

## Abstract

**Objective:**

Cerebrospinal fluid total protein (CSF-TP) levels in adults with posthemorrhagic hydrocephalus (PHH) are poorly studied. The objective of this study was to explore the characteristics of CSF-TP levels in patients with PHH.

**Methods:**

The clinical data of 156 patients with hemorrhagic brain disease were retrospectively studied and divided into PHH and NPHH groups. Single-factor and multi-factor analyses were performed, and the key role of CSF-TP was evaluated using linear analysis.

**Results:**

Among the 156 patients, 85 (54.5%) had PHH and 34 (21.8%) underwent surgeries. Hypertension (*p* = 0.017), days [total fever time when body temperature ≥ 38.5°C (*p* = 0.04)], Glasgow Coma Scale (GCS) score (*p* < 0.001), and time (from the onset of the disease to the obtainment of CSF-TP after lumbar puncture (*p* < 0.001) were important factors for PHH. Logistic regression analysis revealed that GCS score < 8 [odds ratio (OR) = 2.943 (1.421–6.097), *p* = 0.004] and CSF-TP × time ≥ 9,600 [OR = 2.317 (1.108–4.849), *p* = 0.026] were independent risk factors for PHH. All CSF-TP values were averaged every 2 days. CSF-TP was negatively correlated with time. Linear analysis showed that CSF-TP in the PHH group was higher than that in the NPHH group at the same onset time, and that the duration of detectionin the CSF was longer.

**Conclusion:**

Cerebrospinal fluid total protein (CSF-TP) × time ≥ 9,600 and GCS score <8 were independent risk factors for PHH. CSF-TP was higher in the PHH group than in the NPHH group.

## Introduction

The term posthemorrhagic hydrocephalus (PHH) was first proposed by Murtagh and Lehman in 1967. It initially referred to the progressive dilatation of the brain ventricular system in neonatal intraventricular hemorrhage (IVH) (1). Later, the usage of the term expanded to include IVH, intracerebral hemorrhage (ICH), subarachnoid space hemorrhage (SAH), and hydrocephalus caused by traumatic brain injury (TBI) (2, 3). PHH is caused by obstruction of cerebrospinal fluid (CSF) outflow, dysfunction of CSF absorption, or CSF hypersecretion (4). This study focused on adults with PHH. In general, hydrocephalus may be acute (<3 days), subacute (4–13 days), or chronic (>14 days). Most cases become chronic and tend to have a very poor prognosis. Patients typically present with neurological dysfunction, abnormal gait, dysuria, dyschezia, etc. The majority of patients with PHH need shunt surgery to improve their symptoms (5, 6). The occurrence of PHH is directly related to the blood volume of the ventricular system (7). At present, the accepted mechanisms mainly involve blood clot blockage, red blood cell (RBC) degradation products, such as hemoglobin and iron ions, platelet-releasing transforming growth factor, and subarachnoid space fibrosis, caused by transforming growth factor-β1 (TGF-β1) (8). Whether CSF-TP values in bloody CSF are related to PHH currently lacks relevant research.

## Materials and Methods

### Study Design and Population

The objective of this study was to analyze the risk factors for hemorrhagic brain diseases complicated by PHH and explore the relationship between CSF-TP and PHH. This study was approved by the Ethics Committee of Jiangxi Provincial People's Hospital (No.2021-074) and was carried out in strict accordance with the Helsinki Declaration (2013). Relevant medical records were collected retrospectively between January 2013 and October 2020. Univariate and multifactorial analyses of clinical factors were performed to determine the independent risk factor. The relationship between CSF-TP and PHH was evaluated using linear analysis.

#### Inclusion Criteria

1. TBI, SAH, IVH, ICH, etc., clearly diagnosed by CT.
2. Biochemical data of the cerebrospinal fluid were collected during hospitalization.
3. Hospitalization courses or traceable courses are longer than 2 weeks (diagnosis of chronic hydrocephalus is longer than 2 weeks).

#### Exclusion Criteria

1. Hematological diseases, brain tumors, and hydrocephalus were diagnosed before onset.
2. Intracranial infection was diagnosed during the course of the disease.
3. Decompression of bone flap was performed.
4. Concomitant complicated liver, kidney, gastrointestinal, respiratory, cardiovascular, and other diseases that seriously affect safety assessment.
5. Concomitant serious infectious disease.
6. Complications of coagulation dysfunction or long-term use of antiplatelet or anticoagulant drugs.
7. Pregnancy or lactation.

### Treatment

All the patients received standardized nursing care and treatment in the neurosurgery department. Our department had a well-trained professional team who strictly followed the guidelines for neurosurgery treatment formulated by China (9–11). After admission, head CT scans, routine blood tests, biochemical tests, and other related examinations were performed. Medical history of the patients including history of present illness was also recorded. Physical examination by a neurosurgery specialist was performed upon admission. Only patients who gave consent personally or through their families were treated.

### Data Collection and Result Evaluation

Basic information (age, sex, diagnosis, GCS score, etc.) was obtained from the inpatient information management system of our hospital, from which past information (hypertension history, diabetes history, drug history, personal history, etc.), detailed treatment information (onset time, lumbar puncture time, days (total fever time when body temperature ≥ 38.5°C), and laboratory test data (CSF biochemistry, blood routine, etc.) were collected. The diagnosis of PHH was based on the first discharge diagnosis, review results, and the second admission diagnosis. Information collection and statistical analysis were conducted by professional personnel.

### Statistical Analysis

The SPSS software (version 26.0; SPSS Inc., Chicago, IL, United States) was used for the statistical analysis, and the statistical tests were bilateral. Regarding the distribution of CSF-TP, the X-axis referred to the time at which CSF-TP was obtained, where the time of disease onset was set at 0. The Y-axis indicated the CSF-TP levels. Statistical classification variables are expressed as counts and percentages (%), and continuous variables are expressed as mean (x) ± SD. Parametric or nonparametric tests were performed according to whether the data conformed to a normal distribution. Univariate analysis was carried out using the *t*-test or Mann–Whitney *U*-test and chi-square test, and multivariate analysis performed binary logistic regression (odds ratio, OR, >2 was regarded as an independent risk factor). For all the tests, *p* < 0.05 was considered statistically significant.

## Results

**Figure 1:**
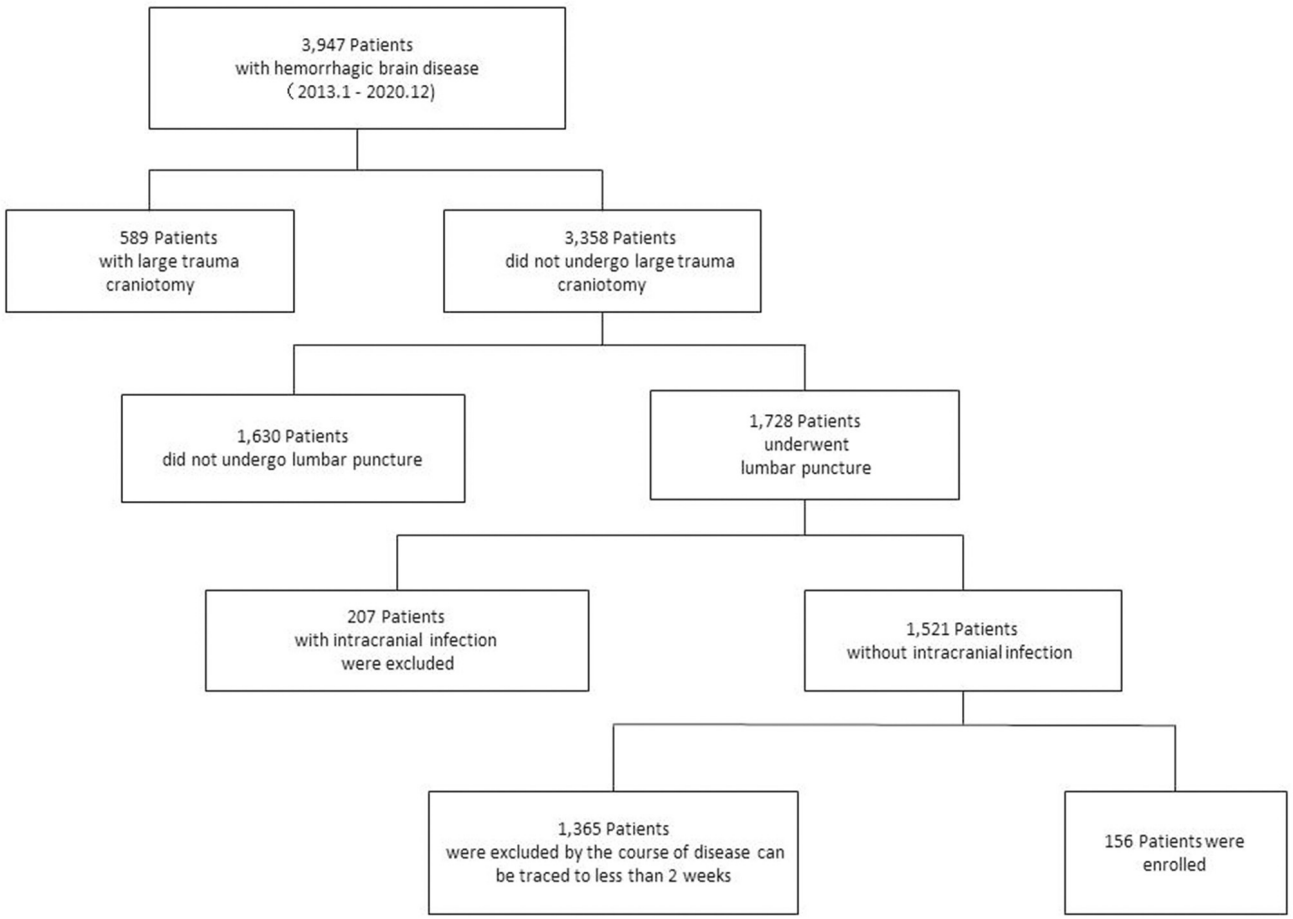
Case inclusion process. A total of 3,947 patients with hemorrhagic brain diseases were treated in our hospital, and 156 patients were selected for retrospective study.

Between January 2013 and December 2020, 3,947 hospitalized patients were screened using a case information system for retrospective analysis. Finally, 156 patients were included in the study based on the inclusion and exclusion criteria. The number of patients in each phase is shown (Figure 1). Of the 156 patients, 85 had PHH and 71 had NPHH. During hospitalization, there were only 84 patients with single CSF test results and 72 patients with ≥2 CSF test results.

### Basic Characters

Data on sex, age, diabetes history, days (total fever time when body temperature ≥ 38.5°C), hypertension history, CSF-TP (latest value taken), and time (from the onset of the disease to the time the CSF-TP was obtained after lumbar puncture) were collected and included in the study (Table 1).

**Table 1:** Univariate analysis of the association between potential risk factors and posthemorrhagic hydrocephalus (PHH).

**Factors**	**Total** **(*n* = 156)**	**PHH** **(*n* = 85)**	**NPHH** **(*n* = 71)**	***P*-value**
Gender	Male	36	36	0.297
	Female	49	35	
Age (year)		62.341 ± 10.858	55.831 ± 13.731	0.005^*^
Diabetes	Yes	6	3	0.681
	No	79	68	
Hypertension	Yes	51	29	0.017^*^
	No	34	42	
Days		5.071 ± 3.693	3.887 ± 3.323	0.040^*^
GCS		8.400 ± 3.367	11.028 ± 3.664	<0.001^*^
CSF-TP (mg/L)		1,159.988 ± 764.418	1,061.127 ± 652.251	0.441
Time		15.624 ± 7.561	9.507 ± 4.581	<0.001^*^

*Results are expressed as number (%) for categorical variables and mean (± SD) for numerical variables*.

*GCS, Glasgow Coma Scale; CSF-TP, cerebrospinal fluid-total protein; PHH, posthemorrhagic hydrocephalus; NPHH, non-PHH*.

* *The difference has statistical significance. The bold values mean that the P-values have statistical significance*.

Variables (age, hypertension, GCS score, time, and days) with statistical significance (*p* < 0.05) in the single factor analysis were analyzed by multiple factors (Hosmer–Lemeshow test, *p* = 0.09) (Table 2).

**Table 2:** Multivariate analysis of important factors.

**Factors**	**Odds ratio (95%CI)**	***P*-value**
Age	1.064 (1.026–1.104)	0.001^*^
Hypertension	1.603 (0.717–3.586)	0.251
Days	1.082 (0.959–1.220)	0.200^*^
GCS	0.802 (0.717–0.896)	<0.001^*^
CSF-TP × time	1.000 (1.000–1.000)	0.001^*^

*GCS, Glasgow Coma Scale; CSF-TP × time, cerebrospinal fluid-total protein × time; CI, confidence interval*.

* *The difference has statistical significance. The bold values mean that the P-values have statistical significance*.

When CSF-TP > 1,200 mg/L and time > 8 days, the rate of PHH increased significantly (>50%) (**Table 4**). CSF-TP × time was statistically significant in the univariate analysis. Therefore, CSF-TP × time was used as a new reference indicator. Then, 1,200 × 8 = 9,600 was taken as the boundary in the multivariate analysis after grouping. The factors were divided into two groups. In binary regression analysis, GCS < 8 [OR = 2.943 (1.421–6.097), *p* = 0.004] and CSF-TP × time (≥9,600) [OR = 2.317 (1.108–4.849), *p* = 0.026] were independent predictors (Table 3).

**Table 3:** Multivariate logistic analysis of the derivation cohort of PHH.

**Factors**	**Odds ratio (95%CI)**	***P*-value**
Age ≥ 60	1.667 (0.791–3.513)	0.179
Hypertension	1.845 (0.892–3.817)	0.099
Days ≥ 5	1.468 (0.685–3.146)	0.324
GCS <8	2.943 (1.421–6.097)	0.004^*^
CSF-TP × time (≥9,600)	2.317 (1.108–4.849)	0.026^*^

*GCS, Glasgow Coma Scale; CSF-TP × time, cerebrospinal fluid-total protein × time; CI, confidence interval*.

* *The difference has statistical significance. The bold values mean that the P-values have statistical significance*.

**Figure 2:**
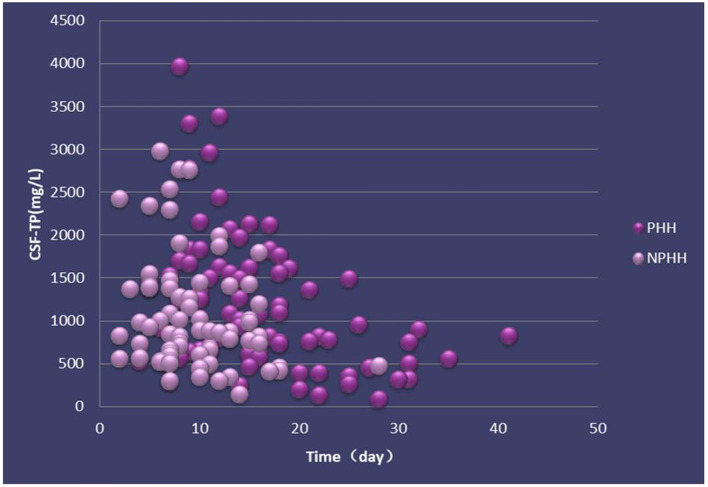
Distribution of CSF-TP in PHH and NPHH groups. X-axis:0 means the beginning of the disease.

**Figure 3:**
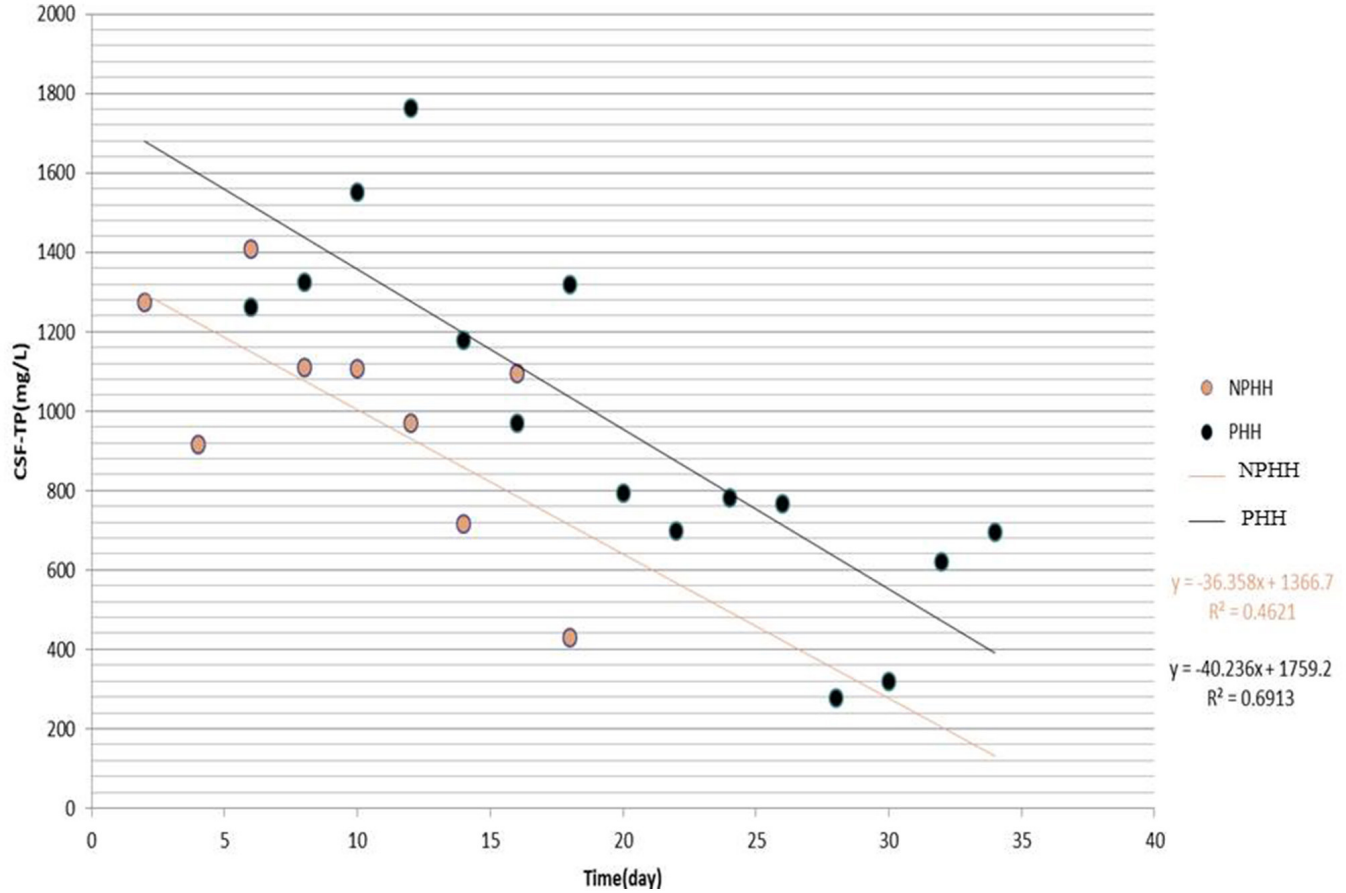
Distribution of CSF-TP after grouping the Time by 2 days. X-axis:0 means the beginning of the disease.

The distribution of CSF-TP of 156 patients in the PHH group was different from that in the NPHH group (Figure 2). The average of CSF-TP values for every 2 days was calculated. The correlation analysis showed that CSF-TP had a near negative correlation with time. Linear analysis was also performed with the following results: NPHH group: *y* = −36.358 × + 1,366.7; PHH group: *y* = −40.236 × + 1,759.2 (Figure 3).

After time was divided into groups (0–4, 5–8, 9–12, 13–16, and >16 days), as well as the CSF-TP values (<400, 400–1,200, and >1,200 mg/L), the number and proportion of complicated PHH cases were calculated (Table 4).

**Table 4:** PHH after grouping CSF-TP and time group.

**Time group**	**PHH**	**CSF-TP group (mg/L)**		
		**<400**	**400–1,200 (%)**	**>1,200 (%)**
0–4	Yes	0	1 (16.7)	0 (0)
	No	0	5 (83.3)	2 (100)
5–8	Yes	1	4 (19.0)	7 (36.8)
	No	1	17 (81.0)	12 (63.2)
9–12	Yes	0	7 (41.2)	14 (73.7)
	No	2	10 (58.8)	5 (26.3)
13–16	Yes	1	12 (63.2)	7 (63.6)
	No	2	7 (26.8)	4 (26.4)
>16	Yes	9	15 (78.9)	7 (100)
	No	0	4 (21.1)	0 (0)

*CSF-TP, Cerebrospinal fluid-total protein*.

*Results are expressed as numbers (%) for categorical variables*.

### Evaluation of Results

**Figure 4:**
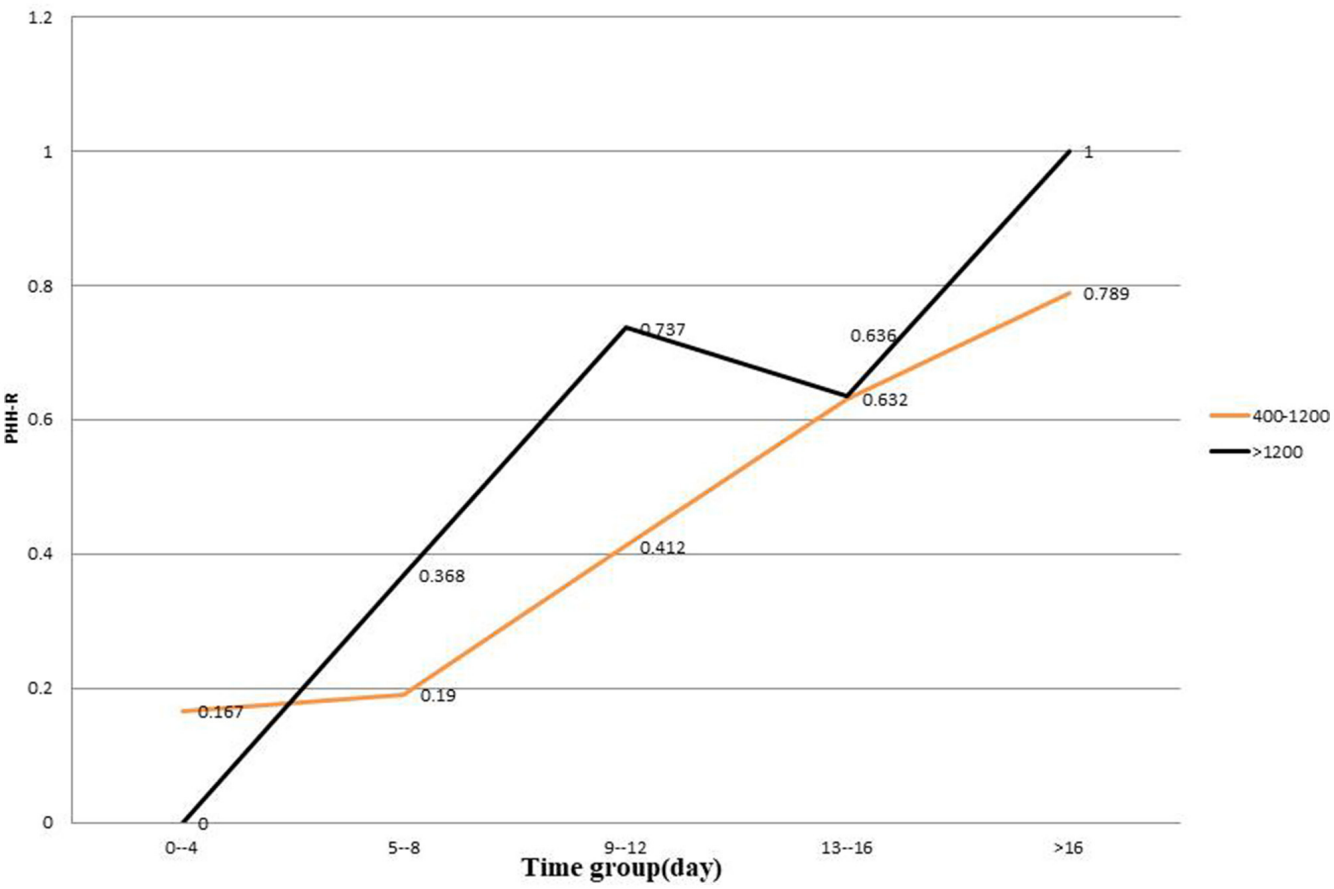
PHH-R in 400–1,200 group and >1,200 group. PHH-R, Rate of posthemorrhagic hydrocephalus.

Age (*p* = 0.005), hypertension history (*p* = 0.017), days (*p* = 0.04), GCS score (*p* < 0.001), and time (*p* < 0.001) were unbalanced variables in the entire cohort. Because the value of CSF-TP changes with time, we analyzed the CSF-TP and time variables as a product of each other, and the results showed that the CSF-TP × time index was a significant factor (*p* = 0.001). After grouping, the binary logistic regression analysis showed that a GCS score <8 [OR = 2.943 (1.421–6.097), *p* = 0.004] and CSF-TP × time (≥9,600) [OR = 2.317 (1.108–4.849), *p* = 0.026] were independent risk factors for PHH. CSF-TP changed gradually with time, and the average TP was obtained by grouping the values every 2 days for statistical linear analysis. The distribution of TP values in the NPHH group was significantly lower than that in the PHH group, with some TP values lower than the normal level, which indicated that the PHH of patients had occurred at this time. The CSF-TP values were divided into two groups (400–1,200 mg/L and >1,200 mg/L). The statistics of the rate of PHH (PHH-R) in the two groups showed that PHH-R increased significantly, and that the >1,200 mg/L group was more obvious (Figure 4).

## Discussion

Posthemorrhagic hydrocephalus (PHH) is a common complication of intracranial hemorrhage that can be secondary to ICH, IVH, TBI, or SAH (5). The mortality rate of the PHH group was higher than that of the NPHH group, and the PHH group generally had a poorer prognosis (12). PHH mainly refers to chronic hydrocephalus, with a time of onset ranging from 2 weeks to 1-month post-injury (12, 13). At present, research on the pathogenesis of PHH focuses on the components of bloody CSF, such as red blood cells, hemoglobin, iron, thrombin, and fibrosis of the subarachnoid space (3, 14, 15). The mass effect of hematoma and ventricular inflammation are the main mechanisms implicated in PHH (16, 17). For patients with intracranial hemorrhage, the development of hydrocephalus is an important predictor of a poor prognosis (18, 19). The progression of hydrocephalus can be used to predict mortality (18, 20). Epidemiologic studies indicate that ventricular dilatation, craniotomy, decompressive craniectomy, and intracranial infection are independent predictors of PHH (12, 21). CSF-TP is the most common CSF biochemical marker, and previous studies on CSF protein in PHH were mainly conducted in pediatric patients, whereas this study mainly involved adult patients. However, studies on the characteristics of CSF-TP in PHH are lacking, and this article focuses on the relationship between CSF-TP changes in bloody cerebrospinal fluid and PHH during hospitalization; therefore, patients with decompressive craniectomy and intracranial infection were excluded.

After IVH, red blood cells in the bloody CSF rarely increase again but gradually lyse. There have been many studies on lysed products; however, there are few reports on the influence of CSF-TP on PHH. CSF-TP is age-dependent, and its concentration in men is slightly higher than in women at 40 mg/L (22). A study on CSF composition changes after IVH showed that CSF-white blood cell (WBC) and CSF-RBC counts increased slightly in the first 3 days then decreased gradually thereafter, beginning on the second day. CSF-TP mainly originates from erythrocyte lysis, platelet release, leukocyte release, and the release of brain tissue from the trauma (23). CSF-WBC is positively correlated with blood loss, which is related to aseptic CSF inflammation in the early stages of the disease (23). A 20-year clinical study suggested that CSF-TP is correlated with WBC and RBC counts (22). However, studies on CSF-TP and routine blood tests in hemorrhagic brain diseases are lacking.

CSF amyloid protein, nerve cell adhesion molecule-L1 (L1CAM), nerve cell adhesion molecule-1 (NCAM-1), and other proteins involved in nerve development are highly correlated with PHH in premature infants (24). Clinically, it is observed that there are high levels of proteins in the cerebrospinal fluid of patients with hydrocephalus, such as thrombopoietin, carbonicanhydrase-I (CA-I) and peroxidase-2 (Prx-2) (25), TGF-β1 (26), S-100protein, glial fibrillary acidic protein (GFAP) (27, 28), neuron-specific enolase (NSE), and vascular endothelial growth factor (29). Absorption decreases after the accumulation of a large number of proteins in the ventricular system (30), leading to increased osmotic pressure and promotion of hydrocephalus (31–33). The contents of I C-terminal propeptide (PICP), N-terminal procollagen III, propeptide (PIIINP), hyaluronic acid (HA), and laminin (LN) in the cerebrospinal fluid of patients with chronic hydrocephalus after craniocerebral trauma were significantly higher than those in the control group (34, 35), suggesting that the subarachnoid space and arachnoid granulations of the patients had obvious fibrosis formation, which may be the pathological basis of chronic hydrocephalus after TBI (34). In a PHH study on children, longitudinal changes in CSF transferring (increased) and ferritin (decreased) levels were associated with ventricular changes and improved neurodevelopmental outcomes (36). In addition, interleukin-10 (IL-10), interleukin-6 (IL-6), interleukin-8 (IL-8), matrix metalloprotein-7 (MMP-7), and matrix metalloprotein-9 (MMP-9) levels were significantly elevated in the CSF protein analysis of PHH (37). The protein components in bloody CSF are complex, and many proteins may play a role in complications associated with PHH (38).

Osmotic pressure changes are caused by the accumulation of a large number of proteins in the cerebrospinal fluid that break the osmotic balance of cerebrospinal fluid circulation and cause hydrocephalus, have attracted much attention (28, 31). Recent studies on human infants have shown that CSF-TP was significantly increased in patients with PHH, but that its effect on osmotic pressure was limited (39). External ventricular drainage, lumbar drainage, and external ventricular drainage (EVD) can reduce the risk of hydrocephalus (12). A clinical comparative study on shunt-dependent chronic hydrocephalus showed that the drainage volume of EVD was not statistically significant, and that the average concentrations of TPd5, TPd11, andTPd14 in shunt-dependent patients were significantly higher than those in non-shunt-dependent patients (*p* < 0.05) (40). The CSF-TP of patients with subarachnoid space hemorrhage (median 130 mg/L) was significantly higher than that of patients without shunt dependence (median 43 mg/L) (41). It is suggested that increase in CSF-TP can predict the occurrence of chronic hydrocephalus after SAH, but there were only 63 patients in this study, including those undergoing decompressive craniectomy.

In conclusion, the authors found that CSF-TP is closely related to PHH. Because the laboratory parameters of CSF are difficult to obtain continuously in a single patient, a large amount of data is needed for analysis. A total of 156 samples that met these requirements were collected. Time was an important factor in PHH, and change in CSF-TP was found to be time-dependent. Although the CSF-TP level decreased after 2 weeks, some of the patients still developed PHH. Therefore, CSF-TP × time was innovatively analyzed as an index, and after setting the reference value at CSF-TP × time ≥ 9,600 (1,200 mg/L × 8) and pooling the data into groups, the multi-factor analysis showed that it is an independent risk factor. Taking the average CSF-TP value every 2 days to establish a linear analysis, it was found that CSF-TP in the PHH group gradually decreased to normal levels within 18 days and lasted for ~35 days, and that CSF-TP in the NPHH group was significantly lower. Therefore, we suspected that patients in the PHH group developed PHH within 18 days. CSF-TP was divided into three groups (<400, 400–1,200, and >1,200 mg/L), and the proportion of complicated PHH every 4 days was observed: <400 mg/L group samples were too less to obtain statistically significant data, and nine cases in the >16-day group had complicated PHH. Finally, it was observed that the proportion of PHH in the 400–1,200 mg/L and >1,200 mg/L groups gradually increased with time, with a more obvious increasing trend for the latter.

There are some limitations to this study. First, this was a retrospective, single-center study, which may have led to unclear data collection and information deviation. Second, other factors that may have affected the results cannot be excluded. Therefore, more clinical data are required, and further prospective randomized trials are needed to confirm the findings of this study.

## Conclusion

Posthemorrhagic hydrocephalus (PHH) is a common complication of neurosurgical hemorrhagic brain disease. There is a lack of research on the pathogenesis and risk factors of CSF-TP. In this study, the clinical data were analyzed using univariate and multivariate analyses, and it was found that GCS score <8 and CSF-TP × time (≥9,600) were important risk factors for PHH, and that CSF-TP in the PHH group was higher than in the NPHH group. Lastly, the time course of changes in CSF-TP levels significantly differed between the two groups.

## Data Availability Statement

The original contributions presented in the study are included in the article/Supplementary Material, further inquiries can be directed to the corresponding author/s.

## Ethics Statement

The studies involving human participants were reviewed and approved by the Biological and Medical Ethics Committee of Jiangxi Provincial People's Hospital (No. 2021-074). Written informed consent for participation was not required for this study in accordance with the national legislation and institutional requirements.

## Author Contributions

RL and HX conceived and supported this research and revised the manuscript. ZW, YC, and XZ designed and conducted the present study, and drafted the manuscript. ZW and FM conducted statistical analysis and interpreted the data. YC and CW collected the primary data. FM and CW completed the follow-up and collected the associated data. ZW and XZ participated in the statistical analysis of data and article writing. All authors of this study met the ICMJE criteria for authorship, made substantial contributions to conception and design, acquisition of data, analysis and interpretation of data, and drafting, critical revision, and final approval of this manuscript.

## Funding

This study was supported by the Beijing Municipal Science and Technology Commission (Grant No: Z181100001518005).

## Conflict of Interest

The authors declare that the research was conducted in the absence of any commercial or financial relationships that could be construed as a potential conflict of interest.

## Publisher's Note

All claims expressed in this article are solely those of the authors and do not necessarily represent those of their affiliated organizations, or those of the publisher, the editors and the reviewers. Any product that may be evaluated in this article, or claim that may be made by its manufacturer, is not guaranteed or endorsed by the publisher.
